# Study design and the estimation of the size of key populations at risk of HIV: lessons from Viet Nam

**DOI:** 10.1186/s12914-018-0141-y

**Published:** 2018-01-30

**Authors:** Ali Safarnejad, Wim Groot, Milena Pavlova

**Affiliations:** 1 Maastricht University, Maastricht Graduate School of Governance, P.O. Box 616, 6200 MD Maastricht, Netherlands; 2 Department of Health Services Research, CAPHRI, Maastricht University Medical Center, Faculty of Health, Medicine and Life Sciences, Maastricht University, Maastricht, Netherlands

**Keywords:** HIV, Population size estimation, Global Health, Data, Vietnam, Surveillance

## Abstract

**Background:**

Estimation of the size of populations at risk of HIV is a key activity in the surveillance of the HIV epidemic. The existing framework for considering future research needs may provide decision-makers with a basis for a fair process of deciding on the methods of the estimation of the size of key populations at risk of HIV. This study explores the extent to which stakeholders involved with population size estimation agree with this framework, and thus, the study updates the framework.

**Methods:**

We conducted 16 in-depth interviews with key informants from city and provincial governments, NGOs, research institutes, and the community of people at risk of HIV. Transcripts were analyzed and reviewed for significant statements pertaining to criteria. Variations and agreement around criteria were analyzed, and emerging criteria were validated against the existing framework.

**Results:**

Eleven themes emerged which are relevant to the estimation of the size of populations at risk of HIV in Viet Nam. Findings on missing criteria, inclusive participation, community perspectives and conflicting weight and direction of criteria provide insights for an improved framework for the prioritization of population size estimation methods.

**Conclusions:**

The findings suggest that the exclusion of community members from decision-making on population size estimation methods in Viet Nam may affect the validity, use, and efficiency of the evidence generated. However, a wider group of decision-makers, including community members among others, may introduce diverse definitions, weight and direction of criteria. Although findings here may not apply to every country with a transitioning economy or to every emerging epidemic, the principles of fair decision-making, value of community participation in decision-making and the expected challenges faced, merit consideration in every situation.

**Electronic supplementary material:**

The online version of this article (10.1186/s12914-018-0141-y) contains supplementary material, which is available to authorized users.

## Background

Estimation of the size of populations at risk of HIV is a key activity in the surveillance of the HIV epidemic and management of the response [1]. Key populations at risk of HIV include, but are not limited to, men who have sex with men, people who inject drugs, and sex workers [2]. Estimation of the size of these key populations is used in three areas of a national HIV response: policy, intervention, and research. In the first area, policy makers use size estimation data to advocate for, mobilize resources for, and prioritize prevention and care programs targeted at key populations at risk of HIV [3, 4]. The second area concerns organizations involved in interventions for key populations at risk of HIV, such as providing clean needle and syringe distribution to people who inject drugs. These organizations need to know the size of their target population in order to plan for, and provide adequate services to particular sub-populations, and monitor the performance of their activities [3, 4]. In the third area, researchers make use of size estimates in evaluating the impact of interventions for key populations at risk of HIV on the overall HIV epidemic, and recommending ways to shift from pilot projects to achieving larger scale coverage of HIV prevention and care programs [4, 5].

Viet Nam is a country with a concentrated HIV epidemic, with an estimated 0.4% prevalence of HIV among the adult population, an incidence of 0.21 per 1000 population, 8600 AIDS-related deaths, and approximately 110,000 people living HIV receiving anti-retroviral treatment in 2015 [6]. HIV prevention, treatment, care and support services are managed nationally by the Viet Nam Administration of HIV/AIDS Prevention and Control in the Ministry of Health with support from multilateral agencies such as the Global Fund, and bilateral programs such as the United States President’s Emergency Plan for AIDS Relief (PEPFAR) [7]. The HIV surveillance system in Viet Nam was established in the early 1990s collecting epidemiological and behavioral data [7, 8]. Among the surveillance activities, a number of estimates of the size of key population at risk of HIV were attempted in Viet Nam [9–11]. Some of these methods estimate the key at-risk population sizes based on a simple multiplier of the general population, using assumptions developed by the Viet Nam HIV estimates and projection technical working group [9]. Other methods use police census information or program data from the Ministry of Labor, Invalid and Social Affairs working with drug users to estimate the size of populations at risk of HIV [9, 10]. More recently, capture-recapture and multiplier methods have been applied to estimate the size of populations at risk of HIV [11, 12]. Still other methods of key at-risk population size estimation with a number of design decisions exist that have not yet been tried in Viet Nam, such as the network scale-up method, the survey-surveillance discrepancy method, or the “never married” method [3, 13, 14].

The concurrent use of multiple methods of size estimation has been justified to validate and interpret the results [15–17]. However, in transitioning economies like Viet Nam, where funding for HIV programs by donors is rapidly decreasing, and the increase in national funding is unable to keep pace to cover the funding gaps, difficult choices are faced in prioritizing HIV surveillance activities such as population size estimation. The limits to the magnitude of resources that can be spent on surveillance, constrains the national HIV program’s ability to conduct population size estimation studies with multiple concurrent methods [18].

Financial cost constraints are not the only force driving decisions in choice of population size estimation method. Decision-makers must also consider the social costs of their decisions related to the methods of surveillance [19, 20], and specifically methods of population size estimation [21–23]. Social costs can include for example perpetuation of stigma and discrimination toward marginalized groups, such as men who have sex with men, and people living with HIV [24]. For example, in examining the link between AIDS stigma and support for name-based reporting, Herek et al. highlight that such policies in surveillance “may evoke anxiety and encounter resistance to the extent that it is perceived as insensitive to – or even fostering – preexisting AIDS stigma” [22].

As decision-makers are being confined to deciding on the “right” size estimation method [25], fair and explicit consideration of a broad set of criteria for prioritization of population size estimation methods becomes imperative. A number of comprehensive approaches exist for health program and research priority setting that define procedures for eliciting criteria and dealing with conflicting criteria [26]. Accountability for Reasonableness (A4R), Combined Approach Matrix (CAM), and Interactive Technology Assessment (iTA) are examples of such approaches [22–24]. Essential National Health Research (ENHR) and the Council on Health Research for Development (COHRED) also provide guidance which has been used in prioritizing health research in developing countries [26–28]. Despite availability of these approaches many health priority setting exercises develop their own, unique methods, because of contextual particularities of priority setting [29]. The Framework for Considering Study Designs for Future Research Needs developed by the Agency for Healthcare Research and Quality (AHRQ) is one framework that specifically considers different study designs for future research needs [30]. The AHRQ framework is intended to standardize the terminology and process in prioritizing health research. The two salient features of the AHRQ framework that distinguish it from the aforementioned health priority setting approaches and make it appropriate for use in this study, are the focus on prioritizing both research and methods of research, and the explicit articulation of criteria related to the selection of research design and methods. In a series of methods papers, AHRQ recommends some criteria and procedures for consistent application in the selection of research design for future research needs [31, 32]. The framework is not intended to be prescriptive, and it lacks a clear description of stakeholder involvement in deliberations, or processes to deal with conflicts and dependencies of criteria. Although the framework has been successfully used in the United States, to our knowledge it has not been applied in developing countries. This framework can potentially be relevant for evaluating the appropriateness of the design of a study focused on size estimation in Viet Nam.

The use of the AHRQ framework is more so appropriate for Viet Nam as it can help to improve accountability and participation in Viet Nam’s HIV Strategy and HIV surveillance activities including population size estimation [33, 34]. Participation of multiple stakeholders (including members of the community who have a stake in the decisions made) to elicit explicit and transparent criteria that play a part in making decisions, is a precondition for a fair priority setting process [35, 36]. Involvement of multiple stakeholders in decision-making processes is grounded in democratic theory [37–39] and the constructivist tradition [40]. The process of research priority setting frequently engages researchers and government but meaningful involvement of other key stakeholders are less frequent [41, 42]. A recent review of 27 national HIV plans found that only 9 plans had specified the community group or civil society involved in the planning process [43]. One reason for limited participation of these stakeholders in decision-making processes is the risk of not reaching consensus, which may lead to less acceptance and trust in the results. Another reason for the limited stakeholders’ participation is that stakeholders, particularly non-expert members of the community, often perceive their values and arguments are not properly considered in national HIV plans.

The AHRQ framework may provide a basis for a fair process of deciding on the HIV surveillance methods including methods of estimation of the size of key populations at risk of HIV. This study aims to explore the extent to which diverse stakeholders involved with HIV surveillance agree with the AHRQ framework. In doing so, we will update the framework, and contextualize it for the problem of deciding on a method of size estimation for key populations at risk of HIV in Viet Nam. An updated framework based on a wide stakeholder involvement will facilitate its use in a fair decision-making process, conforming to the A4R framework [35, 36]. Findings of this study may give other transitioning-economy countries insights into relevant criteria for prioritization of population size estimation methods among other HIV surveillance activities and the role of different stakeholder in that decision-making process.

## Methods

### Study design

We designed a qualitative study to capture various perspectives of the size estimation process, including selection of appropriate method, implementation of the method, and use of the generated evidence. The study took place in 2015 in Ho Chi Minh City and Vinh City. Ho Chi Minh City is a highly urbanized city, with the largest population in Viet Nam (7,123,340 inhabitants in 2009) [44], whereas Vinh City is a provincial city of 230,000 inhabitants and it is considered to be one of the poorest cities in Viet Nam [45]. The data collection method of the study was in-depth interviews with key informants who were previously involved in population size estimation studies.

### Key informants

The study used a purposive, non-random sampling strategy. Since there is no hypothesis being tested and no associated level of confidence in any test results in this study, the number of key informants was not specifically pre-defined. The focus was on reaching as many informants as possible within a pre-defined period. The key informants were selected through personal contacts based on criteria of availability, subject matter knowledge, and representation of the diversity of stakeholders. In each city, we intended to identify one health program manager representing the Provincial AIDS Program, one technical expert from an NGO or research institute involved in surveillance, and one individual from the community of people at risk of HIV in Viet Nam. The motivation behind selecting these three groups was that they encompass the actors who have a stake in the decision-making in surveillance activities, including population size estimations, at the provincial level in Viet Nam. These three groups - representing government, research and development partners, and community members - are referenced by the Greater Involvement of People Living with HIV principle formalized at the 1994 Paris AIDS Summit, and also reflect the membership structure of the Country Coordinating Mechanism of the Global Fund at the central level [46, 47].

As this qualitative study ran concurrently with a size estimation demonstration pilot, participants in that study facilitated the selection of key informants for this study. Following the first interviews, the key informants were asked to nominate other candidates from their organization or network to be interviewed. We asked the key informants to suggest individuals who would be representative of their peers and who would be likely to speak candidly with us. No individuals refused to participate. We completed 16 in-depth interviews in total.

### Data collection

**Fig. 1:**
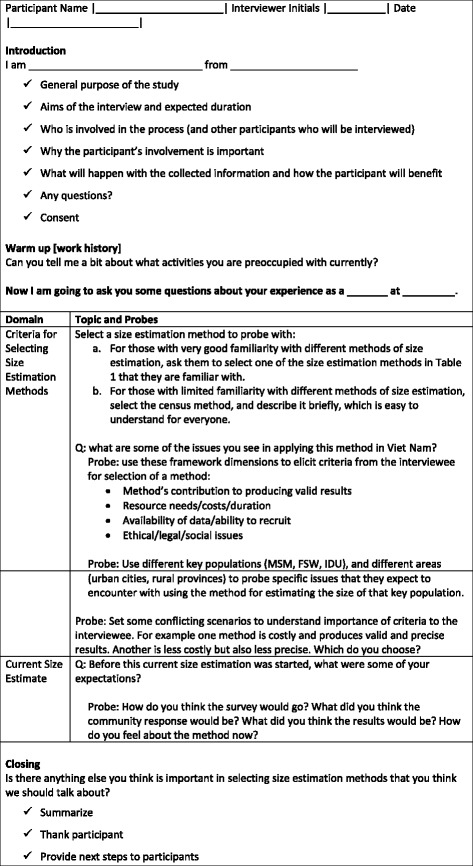
Interview Guide

Verbal and written information about the study were given to each potential key informant. Participation was voluntary and the respondents were informed that they could withdraw at any time and that all data would be treated confidentially. Individual verbal informed consent was obtained from the participant at the beginning of each interview. The survey protocol and instrument material used in this study, and the concurrent population size estimation pilot, were reviewed and approved by the Institutional Review Board of the Hanoi School of Public Health (Hanoi, Viet Nam). An interview guide with open-ended questions aided the focus of the interviews (see Fig. 1 for an outline of the topics and probing questions). There is flexibility in the interview guide to offer space for key informants to raise other issues that they might consider to be pertinent. A table describing the methods of size estimation and the acknowledged strengths and weakness along common criteria established by standards setting bodies, was introduced to the key informants to aid the interviews (see Additional file 1). All interviews were conducted in Vietnamese and later transcribed in English.

The interviews followed an informal format. Pre-defined questions in the interview guide directed the conversation to those topics that matter to the study, while ad hoc questions followed the direction of the conversation. The interviews were conducted while interviewer and key informant were seated at a public or private location chosen by the key informant. The procedures and setting, and the existing relationships of the research team with the key informants made the interviews similar to a ‘conversation with a purpose’ [48, 49]. The approach created an open situation in which experiences and perceptions (positive and negative) could be openly shared, without the key informants fearing they were being too critical. However, all respondents were assured of the confidentiality of the data and that the interviews are intended to be a non-judgmental but formative learning opportunities.

Trained investigators with experience in in-depth interviewing for qualitative research conducted the interviews. Interviews lasted from 30 to 90 min. All interviews were conducted in person and audio recorded (with the key informants’ consent) and were transcribed verbatim. The transcripts were translated to English by the interviewers. Interviews were conducted in August–November, 2015. The authors’ experience and observation as a participant in discussions and decisions on methods of surveillance were also used as an input to the exploration of the research questions in this study.

### Analysis

Transcripts were analyzed according to qualitative research guidelines. Transcripts were read several times by one investigator to search for and code the key informant’s most significant statements pertaining to criteria. The emerging codes were recorded in a codebook, which included a compilation of the codes, illustrative quotes attributed to respondent profiles, and statements that guided the use of the code. Codes that seemed to have similarities were grouped into thematic patterns based on the consensus of all three investigators. Disagreements about grouping of codes into thematic patterns were resolved through a discussion until consensus was reached. There were no occasions that consensus could not be reached. The emerging themes were documented and maintained as a permanent record of our analysis progress. These steps were repeated until no new themes emerged. Criteria for choosing the population size estimation methods were extracted from the emerging themes, arrayed by the profiles of the key informants. This combined process allowed us to compare and contrast themes within and between the different key informant profiles and different sites. Comparison and contrast of views of themes between key informants also involved identifying sources of variation or agreement. Emerging criteria were validated against the criteria in the AHRQ framework.

## Results

In-depth interviews were carried out with 16 key informants. There were five key informants representing the government, three key informants representing NGOs and research institutes, and eight key informants representing the community of people at risk of HIV. There were an equal number of key informants participating from Vinh City and Ho Chi Minh City. Information on location of key informant interviews and their group membership is provided in Table 1.

**Table 1:** Key informants location of interview and group membership

Key informant	Location of interview	Group membership
1	Vinh City	Government
2	Vinh City	Government
3	Vinh City	Government
4	Vinh City	NGO/Research Institute
5	Vinh City	NGO/Research Institute
6	Vinh City	Community
7	Vinh City	Community
8	Vinh City	Community
9	Ho Chi Minh City	Government
10	Ho Chi Minh City	Government
11	Ho Chi Minh City	NGO/Research Institute
12	Ho Chi Minh City	Community
13	Ho Chi Minh City	Community
14	Ho Chi Minh City	Community
15	Ho Chi Minh City	Community
16	Ho Chi Minh City	Community

In the analysis of the data collected in the interviews, 11 themes emerged as having particular relevance to the process of selecting the method for the estimation of the size of populations at risk of HIV. Table 2 summarizes the 11 emerging criteria theme and how they relate to the criteria in the AHRQ framework. In the following sections, for each criteria theme, we present the summary of findings, along with quotations from key informants that express common views or concepts. The criteria themes are ordered alphabetically for easier reference.

**Table 2:** Criteria developed in this study and their relationship to the AHRQ framework criteria

	AHRQ Criteria			
Criteria developed in this study	Validity	Resource use, size, & duration	Availability of data & ability to recruit	Ethical, legal & social issues
Appropriate for the community				●
Community Participation	●		●	●
Feasibility			●	
Duration		●		
Data Validity (Accuracy/Reliability)	●			
Cost/Resources		●		
Confidentiality				●
Impression of Method			●	●
Equity				
Sustainability/Repeatability				
Risk/Uncertainty				

### Appropriate for the community

Key Informants shared several concerns that were grouped under the *appropriate for the community* criterion. Less than half of all key informants interviewed mentioned this criterion (*n* = 7). But within the sub-group of eight key informants, who are also at-risk population community members, the majority mentioned this criterion (*n* = 6). The most common concerns were about methods that make the subjects in size estimation studies feel uncomfortable and stigmatized. For example, strangers calling gay individuals at their home and asking detailed questions about their sexual behavior. Another concern was about the type and value of incentives given to participants in population size studies. One key informant talking about incentives given to people who inject drug to participate in surveys said, “they don’t want mobile phone credit, they want money”. When asked why they want money, the key informant said, “to buy their next dose of drug.” The question of what is *appropriate for the community* to ensure participation in the population size estimation remains a valid criterion to consider alongside others. This criterion is closely linked with the *community participation* criterion (see below), in that the involvement of peers in the studies helps to avoid the situation of participants being called by strangers.

### Community participation

The importance of the participation of the community in size estimation studies was reiterated by most of the key informants (*n* = 13), including three participants from the government, two participants from NGOs and research institutes, and eight participants from the community of people at risk of HIV. They cited examples of recruiting peer-educators to help identify hotspots, building relationships with establishment workers to allow access to interview the at-risk populations, and working with local authorities to triangulate data for improved accuracy. The latter involvement of local authorities also helps to reassure participants in size estimation studies about the legality of the study, which was cited by one key informant as a common concern particularly in provinces. *Community participation* is also a means to ensure learning for the community, which is linked to the sustainability and *repeatability* and long-term cost-effectiveness of the study methods. Five key informants, all of whom came from the community of people at risk of HIV, considered community learning as an important aim of *community participation*.

It is not just the relations and networks that matter in population size estimation studies, but also the reputation of the investigators. The key informants told us that when participants in studies trusted the investigators, they were more forthcoming and honest, and complete in their disclosure. Trusted members of the community are privileged to receive unadulterated information from their peers, which helps to generate accurate and reliable size estimates.

Communities of key populations, like any social networks, are not symmetrically connected, and members of the community vary in the strength of their ties. Local knowledge about the “sociometric stars” (individuals whose high regard among their peers enables them to recruit their peers [50]) and hidden community members increases the speed of reaching the desired number of participants in size estimation studies, as well as the reach of the study to a diverse range of community members. One key informant however, refuted the notion of *community participation* to improve recruitability, saying that “hidden populations never take part in community activities”, so the involvement of community peers and social networks do not amount to universal recruitability.

### Confidentiality

While nine key informants considered confidentiality as a criterion in the selection of methods for size estimation, most of those key informants were at-risk population community members (*n* = 6). The level of confidentiality they mentioned ranged from total anonymity to discretion. One key informant contextualized the confidentiality issue in relation to the level of stigma in the region where studies are conducted: “In Ho Chi Minh City, confidentiality is not a big deal but in non-urban areas where stigma is high, people might not want to leave phone numbers.” Another key informant said that providing personal information had also to do with the self-stigma: “ones who are ‘closed status’ will not provide it”, referring to those who are not open with their friends and family about their homosexuality, HIV status, or other socially stigmatized status. Key informants familiar with self-administered surveys noted that the privacy and confidentiality inherent in self-administered surveys improved the data accuracy by reducing social desirability bias that are more prevalent in face-to-face surveys.

Contrary to the sentiments that *confidentiality* should be a criterion, one key informant experienced in implementing size estimation studies, noted that “people report to local government authorities if they are asked to take part in a survey. They want reassurances”. In other words, attempts to keep the survey confidential are futile, because people are afraid they are doing something illegal. This implies that confidential surveys may conflict with the criterion of *feasibility* (see below).

### Cost/resources

More than one half of the key informants had something to say about costs and resources required for population size estimation (*n* = 9), including five participants from the government, three participants from NGOs and research institutes, and one participant from the community of people at risk of HIV. The views on *cost/resources* varied widely among participants. One thought that cost should be the last criterion, while another placed it as the main criterion, preferring a low-cost method. One key informant addressed the concerns about cost by suggesting: “method should have flexibility to keep costs down by using volunteers”. Another key informant refuted the idea, giving an example of their experience using student volunteers: “One year we used students to do mapping. They could not identify the correct location of drug users or sex workers. Where there were drug users, they said no. Where there were many females but it turns out they were not female sex workers”. The key informant went on to propose enlisting the help of community members, drug users turned peer-educators, in the size estimation studies. Another key informant further qualified this proposal by suggesting that “quality and number of staff with adequate capacity” should be considered when estimating resource needs.

### Data validity

Data validity, expressed as reliability and accuracy of data, was the most frequently cited criterion in considering the methods of population size estimation (*n* = 13). There was little divergence in terms of how accurate the generated data should be. Several key informants talked about “acceptable”, “adequate”, and “reasonable” data validity (*n* = 3). One key informant said, “70 to 80 percent accuracy is good enough; doesn’t need to be 100 percent accurate”. Another key informant affirmed this opinion, saying “just get closer to the truth”.

There was more divergence in how important the data validity criterion is vis-à-vis other criteria. Some key informants considered it a sub-criterion of *cost/resources* (*n* = 4). “Given available resources, we should aim to produce good results. If resources are limited, we should aim to produce adequate results” one key informant explained. Another key informant stated that data accuracy depended on the skills of investigators in the size estimation as well as the involvement of the community.

### Duration

Half of the key informants expressed some preferences - either longer or shorter – for the duration of size estimation studies (*n* = 8). One key informant reasoned that “time required for implementation should be short, so that the estimates can be repeated often for update of the data”, suggesting that repeating the exercise over and over again will reinforce the reliability of the data. A key informant who had also been a participant in a recent size estimation study of men-having-sex-with-men, had a different perspective: “[they] like the quick-to-fill surveys, though it is probably skipping many additional questions that would improve reliability”. Both key informants ultimately agreed that *reliability* was the desired outcome, despite duration of the study.

Other differences on duration were around the accuracy of short duration studies. “Time for census should be increased to identify if a person is from [this province] or here temporarily” said one key informant, while another pointed out: “seasonal nature of sex work makes some methods inaccurate, because of extended time required for the method”. However, they conceded that longer duration studies came at higher *cost* as well. A critical perspective of *duration* as a criterion came from a key informant who considered it “a sub-criterion after considering the urgency of the data needed.”

### Equity

A few key informants made references to the differences in the applicability of the population size estimation methods in different geographical areas: rural versus urban areas (*n* = 4). The most frequent reasons included geographic grouping of key populations in hotspots, more prevalent use of Internet and mobile devices in urban areas, and better roads and access in cities than rural mountainous provinces. Not all opinions however favored urban environments, with one key informant saying: “rural studies are easier; people are more likely to answer honestly”.

Two key informants invoked the differences in the methods’ ability to work for different key populations. Methods that rely on recognition or identification of hotspots were questioned for particular key populations: “Female sex workers are easier to recognize, gather in hotspots; men who have sex with men use social networks, so reach is less costly” one key informant stated.

Age of people at risk of HIV also factored into the size estimation methods’ equitable application to all populations. Community activism is relatively new in Viet Nam, so younger gay men are more involved with the LGBT community and therefore it is easier to recruit their help in size estimation studies when the study aims are aligned with community aims.

### Feasibility

All factors that are external to the methods of size estimation, such as the environment and history, which affect the decision to select one method over another are considered issues of feasibility. Two recurrent external factors mentioned in the interviews were ‘willingness to participate’ and ‘structures in place’. These factors were merged to develop the criterion of *feasibility*. Majority of key informants mentioned this criterion (*n* = 12), including all at-risk population community members who were interviewed (*n* = 8).

For two key informants, the notion of ‘willingness to participate’ stemmed from the at-risk population’s sense of community and civil duty towards that community. That is, the stronger the sense of community among the members, the more willing they would be to participate in the size estimation studies, thus making the study more feasible (see criterion on *Community Participation*). Key informants also noted ‘convenience for participants’ as a factor in determining the willingness to participate (*n* = 4). This convenience was both in terms of how easy the questions in the surveys would be to answer, but also how convenient the process would be for participation. An online survey for example would be easier to organize for participants and investigators, than a face-to-face interview that would require organizing a convenient time and place for both.

Key informants mentioned that ‘stigmatized subjects’ and ‘survey fatigue’ are two deterrents to the participation in size estimation mentioned (*n* = 4). In the former case, one key informant said: “if the theme is sensitive and involves stigma, it is difficult to do”. In the latter case, survey fatigue is a result of a long history and large volume of surveys and surveillance activities – often without the involvement of the community – that has diminished the ability of new surveys to recruit participants, and therefore diminishes the *feasibility* of future size estimation studies.

Key informants frequently talked about ‘structures in place’ that make size estimation studies more feasible (*n* = 8). Key structures elicited in the interviews included technologies, like Internet and mobile network access, to facilitate surveys. Other structures in place included key population gathering hotspots, social networks of key populations, and physical infrastructure such as roads to reach rural mountainous provinces. Experienced investigators were also noted as making a positive contribution to the *feasibility* of study methods. In the absence of these structures in place, the choice of methods for size estimation diminishes along with the *feasibility* of the study methods.

### Impression of method

One of the themes that developed in the interviews, and the third most frequently cited criterion, was around the impressions that the key informants held about the method of size estimation, and how that impression affected their choice of method (*n* = 10). In reference to various methods, the key informants used phrases like “sense of seriousness”, “seems exclusive”, “seems rigorous”, “more professional”, “have confidence in”, “state-of-art”, and “novel”. In probing the key informants, one said “people like things related to technology”, in reference to novel methods of using social media for size estimation. The impression of “exclusiveness” was explained by another key informant as being created by disqualifying some respondents: “I was surprised by the limited number of invitees”, one said, “not like the typical poll created online”.

The most compelling explanation came from a key informant who explained the criterion from the perspective of decision-makers: “simple methods are often seen with skepticism, whereas more complicated methods carry more weight. Perception of people about the method matters. Sometimes people prefer more complicated methods, because it sounds more scientific and so it must produce better results. A method that involves simple counting might be suspected to be too easy to be true. Sometimes, in order to get buy-in, we may need to rely on more complicated methods.”

The impression that the key informants had of a method was clearly a criterion for the selection of that method. Novel methods were positively considered by the key informants. This novelty of method addresses survey fatigue, increases interest and recruitment of participants, and improves acceptance of results by stakeholders. However, two key informants who had been involved in recent size estimations as investigators recalled “the novelty of the method made it a painful process”, and felt “anxious” about getting results. Novel methods also lacked the historical data to validate the reliability of their results.

### Repeatability

A criterion that is closely linked to, but distinct from the *community participation* and *cost* criteria in choice of population size estimation is the *repeatability* of the method. Only one key informant mentioned this criterion. It speaks about sustainability and the long-term cost-effectiveness of a method as the community learns how it works and applies it using volunteers in the community. As the key informant put it, a method that “people can learn and do it later”.

### Risk

An important but seldom mentioned criterion was the dependence of the methods on uncontrollable factors (*n* = 1). This theme emerged from a conversation with one key informant who was involved in a recent respondent driven sampling (RDS) survey, where referrals were trickling in too slowly and jeopardizing the validity of the results, and also increasing the overall cost of running the study. Methods of population size estimation that use RDS carry more uncertainty because they depend on people’s willingness to refer. This uncertainty in the methods’ ability to produce the required results was given a thematic label of *risk*.

## Discussion

We explored the perspectives of multiple stakeholders in Viet Nam who were previously involved in population size estimation studies, on criteria relevant to selecting methods of population size estimation for surveillance of HIV epidemic, and the extent to which these criteria agree with the AHRQ framework for Considering Study Designs for Future Research Needs [30]. Our findings are consistent with the AHRQ framework, but our work further clarifies the dimensions of this framework when applied to population size estimation methods, and extends it to include three newly identified criteria: *repeatability*, *risk*, and *equity*. The latter *equity* criterion was defined in terms of methods that are appropriate for different age groups, at-risk populations, and urban/rural settings. The addition of these criteria to the AHRQ framework will increase its breadth and relevance to the prioritization of methods for estimation of size of populations at risk of HIV.

In addition to the new criteria identified, it is important to note that some other criteria would have been missed if the study did not include a diverse group of stakeholders. While all key informants from the government, NGOs and research institutes considered *cost* an important criterion, only one key informant from the community of people at risk of HIV thought this criterion was important. Conversely, *community participation* was considered a criterion by the majority of key informants, but no key informants from the government, NGOs and research institutes saw it as an opportunity for community learning. Our findings illustrate the dichotomy of views of stakeholders on criteria for prioritization of methods of size estimation, and underlines the importance of an inclusive and interactive process that considers the opinion of technical experts, health managers, but also the community that is the beneficiary of the evidence-based services [51–53]. An important implication of this finding at the national level is the need for inclusive decision-making that involves the community. While participation of community members in strategic planning of the HIV response in Viet Nam is affirmed [33, 34], their participation in technical and scientific decisions like those of population size estimation methods must also be supported. This “democratization of expertise” may well require investments in technical literacy of community based organizations to strengthen their role in decision-making or grass-roots movements for community-driven policies in research, science and technology [54, 55].

Among criteria elicited by multiple key informants from diverse groups, perspectives of how a criterion is defined sometimes diverged significantly. *Feasibility*, for example, is a criterion that is often elicited in research prioritization [27, 28, 41]. In our conversations, key informants from the government, NGOs and research institutes defined *feasibility* in terms of structural enablers in place to support the method, such as mobile telephone technology, roads to get to remote villages, and experienced investigators. We call this the systems perspective of the criteria. Key informants from the community of people at risk of HIV, clarified *feasibility* in terms of the individual recruits’ willingness to participate in the size estimation studies – due to convenience of participation, interest in the novelty of the method and the learning opportunity, and a sense of community or civil duty to their community. We call this perspective the community perspective of the criteria. Another instance in our study where the community and system perspectives are evident, is in the discussions around *duration*. One key informant spoke about duration from the perspective of an individual survey taker (community perspective), while another took the perspective of the entire duration of a size estimation study (system perspective). Our study points out the importance of this dual perspective to help decision-makers derive a more complete and legitimate definition of the criteria.

Moreover, the findings suggest that feasibility of some methods may depend as much on the systems and structures in place, as it does on the strength of ties within the community of participants who are the subjects of the study. In line with the recommendations of Johnston et al. [56], our findings point out a specific need for better evidence about the strength of ties within the community, as an indicator of their willingness to participate in population size estimation studies. The strength of ties can be measured for example by conducting a survey in the community, recruiting participants through RDS, and asking respondents to grade their relationship with the person who referred them using profiles of relationships developed by Spencer and Pahl [57] or using Dunbar’s theoretical boundaries of social contacts [58].

Where there was agreement on the definition of criteria, key informants sometimes differed in how important they considered one criterion vis-à-vis others. In prioritization frameworks, these relative differences are called the weight of the criteria [59, 60]. Another crosscutting theme that emerges in reviewing the criteria elicited in this study is that there were differences among the key informants about the direction of some criteria. These differences were sometimes considerably varying, with some key informants seeing a criterion as a positive factor for selecting a method, and others seeing it in a negative light. Criteria that exhibited these differences in weight and direction include *confidentiality*, *cost/resources*, *data validity*, *duration*, *equity* and *impressions of method*. The weight and direction of criteria affect the priorities in methods of population size estimation when the criteria are applied. A number of structured procedures exist to quantifying the criteria weights and directions of the criteria. Such procedures include discrete choice experiments, conjoint analysis, ranking and rating of criteria [59, 60]. These procedures would be an important addition to the AHRQ framework to prioritize methods of population size estimation.

The findings above on missing criteria, inclusive participation, community perspectives and conflicting weight and direction of criteria, provide insights that help us improve the AHRQ framework in its application to the prioritization of population size estimation methods. These findings underline the importance of inclusion of diverse group of stakeholders, particularly the community of people at risk of HIV. These findings and recommendations are also in line with the expectations of the authors of the AHRQ framework for it to be refined and contextualized in the future [30].

To our knowledge, at the time of this study there is no known application of the AHRQ framework in developing country settings or to HIV surveillance. This study furthers our understanding of methodological issues that may be faced in applying the framework. Comparison of the study findings in Viet Nam, to best practices found in literature, allows us to provide a number of suggestions to clarify the role of stakeholders in the priority setting process:

First, our results showed a number of potential conflicts and dependencies between criteria identified. For example, two key informants in our study had different definitions of the *duration* criterion, but ultimately agreed that *reliability* was the desired outcome. Youngkong et al., who conducted a systematic review of health care priority setting in low-income countries, posit that differences in definitions of criteria may be dependent on culture and perspectives of the stakeholders [61]. They predict that in joint discussions with relevant stakeholders a more suitable set of criteria may be obtained. Guidance on multi-criteria decision-making recommends focusing the group discussion on organizing criteria into a hierarchical structure, and combining criteria when there is potential redundancy and decomposing criteria when alternative definitions of criteria are elicited [62]. This process of representing the decision analysis jointly is believed to have an indirect value in raising consciousness about the root of any conflict [63].

Second, our results showed potentially different weight and directions assigned to the criteria by a group of stakeholders. Kerr and Tindale have discussed the use of a number of approaches to group decision-making [64]. Perez et al. present use of fuzzy set theory to model and deal with vague or imprecise options, alternatives, and opinions of several decision-makers [65]. Shukla and Auriel suggest a framework for conducting criteria weight analysis under multi-stakeholder scenarios, but with an emphasis on transparency, avoidance of conflicts, low cognitive load, and taking into account multiple decision-makers with different perception of criteria [66]. It is the latter approach that we recommend for the management of diverse definitions, weight and directions of criteria when a wider group of decision-makers, including community members, are consulted in decisions on population size estimation methods.

The primary aim of this study, like other qualitative research, is to provide a rich, contextual understanding and not to generalize results, so representative samplings is not as important as the ability of the selected participants to provide their diverse perspectives [67–69]. Nevertheless, the depth and coverage of those perspectives on population size estimation methods is limited in our study by the time allowed with few key informants in few locations, and how the key informants were selected. We tried to overcome the limitation in the external validity of our study by employing the four strategies recommended by Sharan Merriam [70]. These include (1) providing enough details in our study so that readers can determine how closely their situation matches it, (2) using multiple sites to allow for application to a greater range of similar situations, (3) comparing the specific criteria in this study to the broader criteria of health research in the AHRQ framework, and (4) sampling within the key informants to ensure representation of the relevant three stakeholder groups.

The AHRQ framework was selected for this study because it provided us with a standardize terminology, fair process, and basic set of criteria to compare against our findings in Viet Nam. Although there is a lack of application of the framework outside of the United States, an aim of this study was precisely the applicability and relevance of this framework to decisions on methods of size estimation for key populations at risk of HIV in Viet Nam.

Another limitation of this study was that no focus group discussions were conducted. Without a debate to test the strength of opinions of key informants on any particular subject, vis-à-vis their peers, we cannot be certain how strongly individuals believe in their opinions. On the other hand, the in-depth interview format did allow some valid, but less popular, points of view to be exposed. The selection of key informants was through introductions from the seed key informants. It is possible that key informants refer individuals similar to themselves in perspectives and experience. However, private interviews with the key informants, and conducting interviews in two separate cities, help to ensure the independence and trustworthiness of the results.

We intended to identify and interview a diversity of key informants, both geographically and also in their representation of key stakeholder groups. We succeeded in recruiting equal number of participants from Ho Chi Minh City and Vinh City. In terms of representation of the three key stakeholder groups, there were fewer representatives from NGOs and research institutes, and greater representation from the community members, due to their availability at the time of the interviews. This could have led to some skewing of relevant criteria in our results toward the community perspective. However, the comparison of the criteria elicited in this study to the criteria in the AHRQ framework gives some external validation of the results.

In the analysis of the interviews all three investigators were involved in the categorization and thematic grouping of codes. However, only one investigator codified the transcripts. While multiple coders would have added rigor and richness to the results, it would have required far more time to review the transcripts and reconcile the codes generated. Having one investigator coding the transcripts also allowed a more uniform definition of the codes to be applied across all transcripts.

## Conclusion

Findings of this study suggest that exclusion of community members from decision-making around key at-risk population size estimation methods in Viet Nam may be contributing to reduced validity, use, and efficiency in evidence generated from these types of surveillance activity. A wider group of decision-makers, including community members among others, may introduce diverse definitions, weight and direction of criteria. Based on these findings and best practices in the decision-making literature we developed a number of recommendations to update and contextualize the AHRQ framework to decisions around HIV surveillance and population size estimation in Viet Nam.

For Viet Nam, we think the AHRQ framework does not have all the criteria that are relevant to stakeholders, and these criteria should be added and considered in future studies. We also suggest using the dual “systems perspective” and “community perspective” help clarify the different definitions of common criteria. When these two perspectives are conflicting, we recommend a process of organizing criteria into a hierarchical structure jointly with relevant stakeholders, and conducting a criteria weight analysis under a multi-stakeholder scenario.

The lessons from Viet Nam may not apply to every country with a transitioning economy. And the lessons from the HIV response may not apply to every emerging epidemic. However, some of the principles of fair decision making, value of community participation in decision-making and the expected challenges faced, merit considering in every situation.

## Additional file

Additional file 1: Summary of Methods of Estimating Population Size. (DOCX 15 kb)
