# Supplementary material for: Study design and the estimation of the size of key populations at risk of HIV: lessons from Viet Nam
Source: BMC Int Health Hum Rights. 2018 Jan 30;18:7. doi: 10.1186/s12914-018-0141-y (PMC5791336; doi:10.1186/s12914-018-0141-y)
Supplement: Additional file 1: — Summary of Methods of Estimating Population Size. (DOCX 15 kb) [file 12914_2018_141_MOESM1_ESM.docx]

Summary of Methods of Estimating Population Size

| **Method Name** | **Description** | **Strength** | **Weakness** |
| --- | --- | --- | --- |
| Census | Count all members of population | simple to understand | time consuming; costly; validity questionable for mobile or hidden populations; |
| Enumeration | Develop Sampling Frame, then count all in frame | less costly than census; | requires reliable sampling frame (statistical expertise needed); validity questionable for mobile or hidden populations; |
| Capture-Recapture | calculate total based on two independent samples | Easy to use; no statistical expertise required; | Difficult to meet assumption in the field (independent, uncorrelated samples, equal chance of selection, identification of captures, no migration, large sample size) |
| Multiplier | calculate size based on a sub-population census and a sampled proportion of total | Easy to use; no statistical expertise required; Flexible method; | Independent data sources required; alignment of age, geography, and time periods between two sources is difficult; data from existing sources may be inaccurate; |
| Population Survey | direct survey of general population about membership in group being counted | surveys are common, easy to analyze, simple to understand and explain; | difficult to use for stigmatized populations/behaviors; limited to households, schools or institutions in sampling frame; perceptions of confidentiality can distort responses; |
| Network Scale-up | survey of general population about size of their network who belong to group being counted | estimates drawn from general population; may produce more valid responses than estimates drawn from key populations (due to reduced respondent bias); one survey can produce multiple pop estimates; | Difficult to estimate average network size; may not represent sub-groups/behaviors hidden from or stigmatized by general population; |

Source: Adapted from “Guidelines on Estimating the Size of Populations Most at Risk to HIV”. WHO and UNAIDS. 2010.
